# Evaluation of lyophilized *Tanacetum vulgare* extract in intraruminal bolus and granule forms for gastrointestinal nematode control in sheep: An *in vivo* clinical study

**DOI:** 10.14202/vetworld.2025.1991-2001

**Published:** 2025-07-22

**Authors:** Aīda Vanaga, Dace Keidāne, Alīna Kļaviņa, Ivars Lūsis, Aija Mālniece, Dace Bandere, Renāte Teterovska, Ance Bārzdiņa, Oxana Brante, Konstantins Logviss, Oskars Radziņš, Līga Kovaļčuka

**Affiliations:** 1 Latvia University of Life Sciences and Technologies, Faculty of Veterinary Medicine, Clinical Institute, Jelgava, LV–3004, Latvia; 2 Latvia University of Life Sciences and Technologies, Faculty of Veterinary Medicine, Institute of Food and Environmental Hygiene, Jelgava, LV–3004, Latvia; 3 Riga Stradiņš University, Faculty of Pharmacy, Riga, LV-1007, Latvia; 4 Baltic Biomaterials Centre of Excellence, Headquarters at Riga Technical University, Riga LV-1048, Latvia

**Keywords:** fecal egg count, gastrointestinal nematodes, intraruminal bolus, phytotherapy, sheep, *Tanacetum vulgare*

## Abstract

**Background and Aim:**

Gastrointestinal nematode infections have a significant impact on the health and productivity of sheep worldwide. Conventional anthelmintics are facing rising resistance, prompting the need for alternative control strategies. *Tanacetum vulgare* (tansy), a traditionally used antiparasitic herb in Latvia, has shown *in vitro* efficacy but lacks *in vivo* delivery validation. This study aimed to evaluate the *in vivo* antiparasitic efficacy and safety of *T. vulgare* extract administered through novel intraruminal boluses and granules in naturally infected sheep.

**Materials and Methods:**

Thirty female Latvian Darkhead lambs (4–5 months; mean 35 ± 0.8 kg) were randomly assigned to five groups: Two bolus groups (A and B), one granule group, and two controls (negative and positive). Groups A and B received intraruminal boluses with different lyophilized extract formulations, the granule group received powdered *T. vulgare* mixed with feed, and the positive control received levamisole. Fecal egg counts (FECs), clinical signs, and hematological and biochemical parameters were assessed over 56 days. Disintegration testing simulated rumen conditions.

**Results:**

No adverse clinical or physiological effects were observed. Bolus groups exhibited a more rapid and consistent reduction in strongylid FECs compared to the granule and negative control groups. On day 56, egg counts decreased to 325–358 eggs per gram (EPG) in bolus and granule groups, compared to 533 EPG in the negative control. Hematological and biochemical parameters remained within reference ranges.

**Conclusion:**

*T. vulgare* extract administered through intraruminal bolus was safe, sustained animal health, and effectively reduced gastrointestinal nematode burden. Granules were less effective, potentially due to reduced palatability. Bolus-based phytotherapy may serve as a sustainable, prophylactic alternative to conventional anthelmintics.

## INTRODUCTION

Gastrointestinal nematodes, such as *Trichostro-ngylus* spp., *Ostertagia* spp., and *Haemonchus contortus*, are among the most prevalent parasitic threats affe-cting sheep in Latvia and throughout Europe [1–4]. These infections compromise host immunity and adversely impact animal health, nutrition, and overall flock productivity. Clinically, parasitic burdens are often associated with hematological and biochemical disturbances [5, 6] and, in advanced cases, can result in diarrhea, submandibular edema, anemia, and even mortality [6–8].

At present, the control of these nematodes relies heavily on synthetic anthelmintics such as ivermectin, albendazole, and levamisole. However, indiscriminate and prolonged use of these agents has accelerated the emergence of anthelmintic resistance worldwide [2–4, 9–11]. In Latvia, recent surveys have confirmed both the predominant parasite species in local sheep flocks and the increasing incidence of resistance to commonly used anthelmintics [1].

Consequently, there is growing interest in exploring alternative control strategies, particularly phytotherapeutic agents, for the prevention and mitigation of gastrointestinal nematode infections [12–16]. Several medicinal plants native to Latvia – *Artemisia absinthium*, *Artemisia vulgaris*, *Calluna vulgaris*, and C*ichorium intybus*, among others – have demonstrated promising anthelmintic proper- ties through traditional use and experimental validation [17]. Notably, *in vitro* study by Kļaviņa *et al*. [18] has shown that leaf extracts of *Tanacetum vulgare* (commonly known as tansy) possess strong ovicidal and larvicidal activity. Despite these findings, there is a lack of published *in vivo* studies evaluating intraruminal bolus formulations of *T. vulgare* or comparing their efficacy to established treatments such as levamisole.

Despite extensive reliance on synthetic anthelmintics for the control of gastrointestinal nematodes in small ruminants, the global rise in drug resistance poses a serious threat to sustainable livestock production. In Latvia and other parts of Europe, resistance to commonly used anthelmintics such as ivermectin, albendazole, and levamisole has been increasingly reported, necessitating the exploration of alternative control measures. Phytotherapeutic agents derived from medicinal plants are gaining recognition as potential substitutes due to their bioactivity and lower environmental impact. *T. vulgare*, commonly known as tansy, is traditionally recognized for its antiparasitic effects and has demonstrated significant ovicidal and larvicidal activity *in vitro*. However, its *in vivo* efficacy remains underexplored, particularly with regard to controlled delivery systems that can ensure sustained therapeutic activity in ruminants.

Notably, there is a lack of studies evaluating the administration of *T. vulgare* in intraruminal bolus form – a promising delivery method that may overcome challenges related to palatability and inconsistent dosing associated with feed-based or aqueous extract delivery. Furthermore, no prior research has compared the antiparasitic efficacy of such phytotherapeutic boluses against established synthetic anthelmintics like levamisole under field-relevant conditions in sheep. This represents a significant gap in the development and application of botanical anthelmintics tailored for ruminant health management.

This study aimed to evaluate the *in vivo* antiparasitic efficacy and safety of *T. vulgare* leaf extract administered through two innovative formulations: 3D-printed intraruminal boluses and feed-based granules. The specific objectives were to: (1) compare the antiparasitic effects of these delivery systems on naturally occurring gastrointestinal nematode infections in sheep; (2) assess changes in fecal egg counts (FECs) over time; (3) monitor hematological and biochemical parameters to evaluate safety and physiological impact; and (4) compare the performance of phytotherapeutic formulations with a conventional anthelmintic (levamisole). By addressing these objectives, the study provides a foundational evaluation of a novel delivery strategy for herbal antiparasitic agents, contributing to the broader search for sustainable parasite control solutions in sheep production systems.

## MATERIALS AND METHODS

### Ethical approval

The Committee for the Protection of Animals Used for Scientific Purposes of the Food and Veterinary Service of the Republic of Latvia approved this study (No. 143/2023, June 08, 2023). The study adhered to ARRIVE 2.0 guidelines for an *in vivo* study.

### Study period and location

The experiment was conducted from October 2023 to January 2024 under controlled environmental conditions at the Faculty of Veterinary Medicine and the Clinical Research Center, Latvian University of Life Sciences and Technologies.

### Animal selection and housing conditions

A total of 30 naturally infected female Latvian Darkhead breed lambs (4 months old; average weight: 35 ± 0.8 kg) were enrolled in a prospective, block-randomized, and double-blinded clinical trial. Animals were stratified based on parasite load and body weight, then randomly allocated into treatment groups through a lottery system. The randomization process ensured blinding of both participants and outcome assessors. All lambs were sourced from a pasture-based partner farm and subsequently relocated to the Clinical Research Center, where they were housed in pens under regulated conditions. Animals were provided *ad libitum* access to hay, mineral-enriched commercial feed (21.9% protein), and water through automated feeders.

### Experimental design and group allocation

Following a 14-day adaptation period (days −14–0), animals were randomly assigned to five groups (n = 6 per group): Group A (bolus with lyophilized *T. vulgare* extract), Group B (alternative bolus formulation), Granule group (feed-based *T. vulgare* granules), N control (negative control), and P control (positive control, treated with levamisole) (Table 1). The experimental phase spanned from day 0 to day 56, concluding with humane euthanasia.

**Table 1:** Experimental groups of the research.

No.	Group	Animals	Description
1	A bolus	6	3D printed intraruminal bolus filled with *T. vulgare* extract lyophilizate
2	B bolus	6	3D printed intraruminal bolus filled with *T. vulgare* extract lyophilizate of different composition
3	Granules	6	*T. vulgare* granules
4	N control	6	Lambs that did not receive granules or deworming drugs
5	P control	6	Lambs dewormed with single dose of levamisole 5 mg/kg subcutaneous injection

*T. vulgare*=*Tanacetum vulgare*

### Clinical evaluation and sampling schedule

Health assessments were conducted twice during quarantine and on days 0, 14, 28, 42, and 56. Parameters included rectal temperature, heart and respiratory rate, mucous membrane color, and weight gain (assessed using Meier-Brakenberg WA 300 electronic scales (Meier-Brakenberg GmbH & Co, Germany). Fecal samples, blood for hematological and biochemical analyses, and clinical observations were collected at each time point.

### Hematological and biochemical assessments

Jugular blood samples were collected on days 0, 14, 28, 42, and 56. Samples for hematology were drawn into 3 mL ethylenediaminetetraacetic acid tubes (Vacutest Kima S.r.l., Italy), while those for biochemistry were collected in 6 mL non-anticoagulant tubes. Hematological parameters analyzed included red blood cells (RBC), white blood cells (WBC), hemoglobin (HGB), hematocrit (HCT), mean corpuscular volume, mean corpuscular HGB, mean corpuscular HGB concentration, red cell distribution width, platelet count, and mean platelet volume using the Exigo Eos Vet analyzer (Boule Medical, Sweden). Blood smears were prepared and assessed under a Nikon Eclipse 80i microscope (Nikon Corporation, Japan) for WBC differential and morphology. Biochemical parameters, including urea, creatinine, bilirubin, aspartate aminotransferase (AST), lactate dehydrogenase, gamma-glutamyl transferase, creatine kinase, total protein (TP), albumin (ALB), phosphorus, calcium (Ca), magnesium, triglycerides, cholesterol, sodium (Na), and potassium, were evaluated using a Mindray BS-200E analyzer (Shenzhen Mindray Bio-Medical Electronics Co., China). All instruments were calibrated and quality-controlled daily.

### Parasitological examination

Fecal samples were obtained rectally using polyethylene gloves and immediately analyzed using the McMaster method (sensitivity: 50 eggs per gram [EPG]). Samples were collected on day 0 and subsequently on days 14, 28, 42, and 56.

### Plant material collection and extract preparation

**Figure 1:**
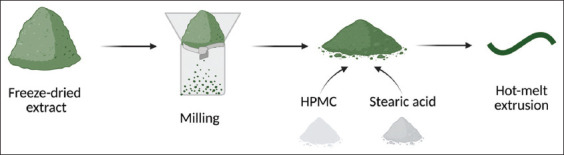
Extract preparation.

*T. vulgare* leaves were hand-harvested in August 2023 from Allaži Parish, Latvia, and identified by Prof. Dr. pharm. D. Bandere. Leaves were shade-dried and stored in paper bags. Extraction was done through maceration in 50% ethanol (1:10 w/v) for 2 h using an orbital shaker, followed by filtration and rotary vacuum evaporation. The semi-solid extract was lyophilized at −80°C and 0.05 mbar for 24 h using a Zirbus Technologies VaCo 2.0 freeze dryer (Zirbus Technologies GmbH, Germany) (Figure 1).

### Formulation of boluses and granules

**Figure 2:**
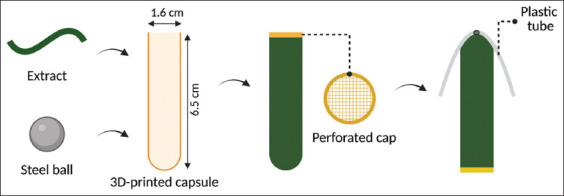
3D boluses design.

3D-printed boluses were created using BioMed Amber Resin and a DLP 3D printer (Asiga pro 4K 80 (Asiga, Australia)). Each cylindrical bolus (6.5 cm × 1.6 cm) featured a perforated cap and was fitted with a steel core and wing for retention and radiographic traceability. The bolus contents were prepared through hot-melt extrusion (HME) of *T. vulgare* extract blended with hydroxypropyl methylcellulose, stearic acid, or polyethylene glycol 300. Each bolus averaged 17.8 g in weight and contained 4 g of extract (Figure 2). Disintegration tests were performed in phosphate buffer (pH 6.8) at 38.0°C.

Granules were made by mixing milled *T. vulgare* leaves with Ca hydrogen phosphate (1:1), then compacted into flakes using a roller compactor.

### Statistical analysis

Data were analyzed using repeated-measures twoway analysis of variance in Stata BE 17.0 (StataCorp LP, 4905 Lakeway Drive, College Station, TX, USA). Sample size (n = 30) was justified using G*Power 3.1.9.6 with f = 0.5, α = 0.05, power = 0.8, and five groups across 5 time points. The model accounted for treatment, time, and their interaction. Tukey’s *post hoc* tests were used for multiple comparisons. Significance was set at p < 0.05.

## RESULTS

### Clinical health and body weight changes

**Figure 3:**
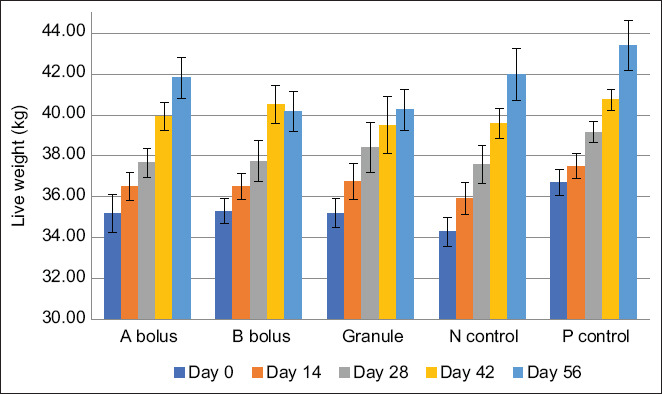
The mean live weight of sheep in the experimental groups over the 56-day period.

All sheep remained clinically healthy throughout the study, showing no signs of distress or disease. There were no statistically significant differences in clinical parameters, rectal temperature, heart rate, respiratory rate, and mucous membrane color, across groups or individuals (p > 0.05). Weight gain was observed in all experimental groups over the 56-day period (Figure 3), with the highest recorded in the P control group. In contrast, the granule group exhibited reduced weight gain, potentially due to lower feed intake caused by the unpalatable nature of *T. vulgare*, despite molasses being added to improve flavor. This likely resulted in a reduced dosage of the active extract.

### Hematological profile and trends

Blood samples collected on days 0, 14, 28, 42, and 56 revealed that hematological values remained within physiological ranges in all treatment groups (Table 2). Regression analysis revealed elevated RBC regression coefficients in Groups A and B at baseline; however, these differences were not statistically significant during the study (Table 3). Group A and P had higher HGB and HCT coefficients early on, but these differences diminished after day 28. Group B showed a higher WBC regression coefficient than the N control on day 14 (p < 0.1) and significantly so on day 28 (p < 0.05). Eosinophil coefficients remained stable across groups. In the granule group, monocyte coefficients increased to 0.18 by day 56, indicating mild monocytosis. Lymphocyte coefficients rose in Group B on days 28 and 42, with no significant trends in the granule group.

**Table 2:** Hematological parameters before and during the research.

Parameters	Time	Experimental group				

		A bolus	B bolus	Granules	N control	P control
RBC (10^12^/L) (R = 6.3–14.6)	Before	10.33 ± 0.48	10.62 ± 0.36	9.57 ± 0.46	9.15 ± 0.24	10.02 ± 0.34
	14 days	10.34 ± 0.39	10.80 ± 0.35	10.32 ± 0.40	9.61 ± 0.26	11.16 ± 0.28
	28 days	10.13 ± 0.32	10.60 ± 0.38	10.02 ± 0.33	9.82 ± 0.37	10.42 ± 0.41
	42 days	10.24 ± 0.40	10.74 ± 0.50	10.53 ± 0.72	9.84 ± 0.44	10.44 ± 0.17
	56 days	10.17 ± 0.47	11.12 ± 0.29	10.23 ± 0.49	10.21 ± 0.27	10.90 ± 0.21
HGB (g/dL) (R = 9.1–16)	Before	11.25 ± 0.54	10.74 ± 0.45	10.27 ± 0.35	9.68 ± 0.20	10.75 ± 0.31
	14 days	11.23 ± 0.53	11.15 ± 0.28	11.10 ± 0.30	10.25 ± 0.25	12.05 ± 0.28
	28 days	10.87 ± 0.46	10.86 ± 0.33	10.93 ± 0.28	10.63 ± 0.37	11.10 ± 0.53
	42 days	11.37 ± 0.46	11.60 ± 0.41	11.58 ± 0.68	10.67 ± 0.47	11.32 ± 0.15
	56 days	11.60 ± 0.64	11.77 ± 0.21	11.20 ± 0.67	11.03 ± 0.24	12.03 ± 0.22
HCT (%) (R = 26–46)	Before	32.03 ± 1.39	29.88 ± 1.00	28.82 ± 1.22	26.88 ± 0.75	30.58 ± 0.89
	14 days	32.25 ± 1.41	31.25 ± 0.90	31.90 ± 1.17	29.23 ± 0.92	34.35 ± 0.86
	28 days	31.28 ± 1.41	30.33 ± 0.91	31.50 ± 1.12	30.35 ± 1.29	31.67 ± 1.53
	42 days	32.65 ± 1.52	31.83 ± 1.74	33.62 ± 2.38	30.45 ± 1.51	32.65 ± 0.70
	56 days	32.33 ± 1.94	33.08 ± 0.76	32.68 ± 2.00	31.08 ± 0.82	34.12 ± 0.88
WBC count (10^9^/L) (R = 4.1–12.1)	Before	7.93 ± 0.72	8.94 ± 0.73	9.03 ± 0.83	8.15 ± 0.58	7.23 ± 0.31
	14 days	7.85 ± 0.64	9.63 ± 1.07	8.67 ± 0.95	7.55 ± 0.50	6.92 ± 0.30
	28 days	8.15 ± 0.75	9.50 ± 0.59	8.88 ± 0.52	7.50 ± 0.48	6.85 ± 0.37
	42 days	7.77 ± 0.61	9.08 ± 0.35	9.38 ± 1.53	7.45 ± 0.53	6.78 ± 0.17
	56 days	8.24 ± 0.60	8.85 ± 0.59	7.58 ± 0.74	7.80 ± 0.41	6.78 ± 0.16
Eosinophils (10^9^/L) (R = 0–4.6)	Before	0.11 ± 0.03	0.09 ± 0.07	0.11 ± 0.06	0.02 ± 0.01	0.10 ± 0.03
	14 days	0.11 ± 0.04	0.35 ± 0.17	0.07 ± 0.04	0.14 ± 0.04	0.15 ± 0.03
	28 days	0.14 ± 0.05	0.11 ± 0.05	0.15 ± 0.06	0.10 ± 0.07	0.08 ± 0.02
	42 days	0.15 ± 0.08	0.14 ± 0.07	0.09 ± 0.03	0.17 ± 0.05	0.10 ± 0.04
	56 days	0.26 ± 0.08	0.18 ± 0.04	0.31 ± 0.09	0.17 ± 0.07	0.13 ± 0.07
Monocytes (109/L) (R = 0–0.5)	Before	0.17 ± 0.03	0.22 ± 0.05	0.18 ± 0.03	0.23 ± 0.07	0.11 ± 0.02
	14 days	0.24 ± 0.04	0.13 ± 0.02	0.30 ± 0.08	0.11 ± 0.01	0.19 ± 0.05
	28 days	0.26 ± 0.05	0.17 ± 0.03	0.24 ± 0.06	0.22 ± 0.05	0.25 ± 0.02
	42 days	0.29 ± 0.08	0.20 ± 0.05	0.36 ± 0.16	0.24 ± 0.05	0.27 ± 0.06
	56 days	0.25 ± 0.02	0.21 ± 0.05	0.36 ± 0.08	0.18 ± 0.03	0.15 ± 0.03
Lymphocytes (10^9^/L) (R = 1.3–9.1)	Before	4.34 ± 0.22	4.48 ± 0.63	4.45 ± 0.46	3.42 ± 0.29	4.05 ± 0.47
	14 days	4.37 ± 0.32	4.59 ± 0.45	5.43 ± 0.64	3.91 ± 0.35	4.19 ± 0.14
	28 days	4.33 ± 0.44	5.53 ± 0.54	5.00 ± 0.65	4.24 ± 0.40	4.00 ± 0.25
	42 days	4.81 ± 0.32	5.60 ± 0.41	4.94 ± 0.62	4.31 ± 0.49	4.65 ± 0.31
	56 days	5.07 ± 0.74	5.80 ± 0.39	4.92 ± 0.61	5.16 ± 0.54	4.53 ± 0.26

R=Range, RBC=Red blood cells, HGB=Hemoglobin, HCT=Hematocrit, WBC=White blood cells

**Table 3:** Regression coefficients of hematological parameters.

Parameters	Time	Experimental group					Intercept	Time	Treatment × Time

		A bolus	B bolus	Granules	N control	P control			
RBC (10^12^/L)	Before	1.18**	1.47**	0.42^NS^	Ref.	0.87^NS^	9.15***	**	NS
	14 days	0.73^NS^	1.19**	0.71^NS^	Ref.	1.55***	9.61***		
	28 days	0.30^NS^	0.77^NS^	0.19^NS^	Ref.	0.60^NS^	9.82***		
	42 days	0.40^NS^	0.90^NS^	0.69^NS^	Ref.	0.60^NS^	9.84***		
	56 days	−0.04^NS^	0.91*	0.02^NS^	Ref.	0.69^NS^	10.21***		
HGB (g/dL)	Before	1.57***	1.06*	0.58^NS^	Ref.	1.07*	9.68***	***	NS
	14 days	0.98*	0.90*	0.85*	Ref.	1.80***	10.25***		
	28 days	0.23^NS^	0.23^NS^	0.30^NS^	Ref.	0.47^NS^	10.63***		
	42 days	0.70^NS^	0.93^NS^	0.92^NS^	Ref.	0.65^NS^	10.67***		
	56 days	0.57^NS^	0.73^NS^	0.17^NS^	Ref.	1.00^NS^	11.03***		
HCT (%)	Before	5.15***	3.00*	1.93^NS^	Ref.	3.70**	26.88***	***	NS
	14 days	3.02*	2.02^NS^	2.67*	Ref.	5.12***	29.23***		
	28 days	0.93^NS^	−0.02^NS^	1.15^NS^	Ref.	1.32^NS^	30.35***		
	42 days	2.20^NS^	1.38^NS^	3.17^NS^	Ref.	2.20^NS^	30.45***		
	56 days	1.25^NS^	2.00^NS^	1.60^NS^	Ref.	3.03^NS^	31.08***		
WBC count (10^9^/L)	Before	−0.22^NS^	0.79^NS^	0.88^NS^	Ref.	−0.92^NS^	8.15***	NS	NS
	14 days	0.30^NS^	2.08*	1.12^NS^	Ref.	−0.63^NS^	7.55***		
	28 days	0.65^NS^	2.00**	1.38*	Ref.	−0.65^NS^	7.50***		
	42 days	0.32^NS^	1.63^NS^	1.93^NS^	Ref.	−0.67^NS^	7.45***		
	56 days	0.44^NS^	1.05^NS^	−0.22^NS^	Ref.	−1.02^NS^	7.80***		
Eosinophils (10^9^/L)	Before	0.09^NS^	0.07^NS^	0.09^NS^	Ref.	0.08^NS^	0.02^NS^	*	NS
	14 days	−0.03^NS^	0.20*	−0.07^NS^	Ref.	0.01^NS^	0.14^NS^		
	28 days	0.04^NS^	0.01^NS^	0.05^NS^	Ref.	−0.02^NS^	0.10*		
	42 days	−0.02^NS^	−0.03^NS^	−0.09^NS^	Ref.	−0.07^NS^	0.17***		
	56 days	0.09^NS^	0.00^NS^	0.14^NS^	Ref.	−0.04^NS^	0.17**		
Monocytes (10^9^/L)	Before	−0.06^NS^	−0.00^NS^	−0.05^NS^	Ref.	−0.12*	0.23***	NS	NS
	14 days	0.13*	0.03^NS^	0.20***	Ref.	0.08^NS^	0.10**		
	28 days	0.04^NS^	−0.05^NS^	0.02^NS^	Ref.	0.03^NS^	0.22***		
	42 days	0.05^NS^	−0.04^NS^	0.13^NS^	Ref.	0.03^NS^	0.24**		
	56 days	0.07^NS^	0.03^NS^	0.18**	Ref.	−0.03^NS^	0.18***		
Lymphocytes (10^9^/L)	Before	0.92^NS^	1.06^NS^	1.03*	Ref.	0.62^NS^	3.42***	***	**
	14 days	0.46^NS^	0.68^NS^	1.52**	Ref.	0.28^NS^	3.91***		
	28 days	0.09^NS^	1.29*	0.76^NS^	Ref.	−0.24^NS^	4.24***		
	42 days	0.50^NS^	1.29*	0.62^NS^	Ref.	0.34^NS^	4.31***		
	56 days	−0.09^NS^	0.64^NS^	−0.24^NS^	Ref.	−0.63^NS^	5.16***		

Ref.=Reference group, NS=Not significant,

* p < 0.1,

** p < 0.05,

*** p < 0.01. RBC=Red blood cells, HGB=Hemoglobin, HCT=Hematocrit, WBC=White blood cells

### Biochemical profile and liver function markers

Biochemical parameters (Tables 4 and 5) indicated that all groups had subnormal TP levels at baseline, likely due to strongylid infections. TP normalized by day 28 in most groups but remained low in some. ALB was below normal at baseline in Groups B and granules, with transient decline noted in Group B on day 28. AST levels were initially normal but fell below the reference range in the granule, N, and P control groups by day 42 and in Group B by day 56. Urea and creatinine levels remained within reference values. TP and ALB regression coefficients were lower in Groups B, granules, and P, with no significant treatment-related changes except a mild shift in the P group. Elevated AST in Group A on day 42 may reflect outlier data from one subject. Although urea levels dipped slightly on days 42 and 56, changes were not significant. Group B’s creatinine regression coefficient was 35.9 (p < 0.01) at baseline but remained stable throughout the study.

**Table 4:** Blood biochemical parameters before and during the research.

Biochemical parameters	Time	Experimental group				

		A bolus	B bolus	Granules	N control	P control
TP (g/L) (R = 59–86)	Before	56.25 ± 1.73	51.33 ± 4.24	51.46 ± 4.33	55.95 ± 1.82	53.73 ± 2.98
	14 days	57.98 ± 2.37	57.17 ± 2.53	60.34 ± 1.96	61.35 ± 2.44	61.55 ± 2.06
	28 days	59.46 ± 1.77	55.41 ± 4.43	55.02 ± 3.95	60.98 ± 1.35	59.90 ± 1.95
	42 days	59.45 ± 0.82	54.03 ± 3.45	54.13 ± 5.23	56.22 ± 5.17	58.63 ± 1.35
	56 days	59.38 ± 3.06	46.01 ± 4.67	53.87 ± 5.86	52.26 ± 5.81	57.57 ± 3.19
ALB (g/L) (R = 25–45)	Before	31.13 ± 2.28	22.55 ± 1.37	22.37 ± 2.14	29.31 ± 1.87	28.14 ± 2.26
	14 days	26.49 ± 1.31	25.86 ± 1.47	26.44 ± 0.94	26.84 ± 0.79	28.01 ± 0.96
	28 days	30.43 ± 0.88	28.44 ± 2.18	27.40 ± 1.66	30.38 ± 0.93	31.60 ± 0.90
	42 days	30.84 ± 0.66	28.03 ± 1.70	28.14 ± 2.36	29.11 ± 2.24	31.40 ± 0.53
	56 days	30.83 ± 1.45	24.39 ± 2.05	28.33 ± 2.83	27.76 ± 3.09	31.91 ± 1.81
AST (U/L) (R = 69–242)	Before	93.00 ± 8.74	90.50 ± 10.96	81.83 ± 9.70	85.33 ± 5.39	82.00 ± 6.80
	14 days	97.00 ± 5.63	95.50 ± 6.86	84.33 ± 4.15	83.67 ± 6.30	83.50 ± 4.02
	28 days	116.33 ± 5.98	105.00 ± 7.34	110.67 ± 27.52	103.50 ± 6.48	97.67 ± 3.11
	42 days	88.50 ± 2.86	70.83 ± 6.91	61.83 ± 9.69	58.67 ± 6.82	68.50 ± 6.18
	56 days	90.50 ± 8.66	63.33 ± 9.95	75.33 ± 12.80	71.50 ± 11.49	79.67 ± 6.45
Urea (mmol/L) (R = 2.5–12.5)	Before	5.71 ± 0.20	4.21 ± 0.42	3.48 ± 0.36	4.49 ± 0.36	4.24 ± 0.45
	14 days	3.95 ± 0.29	4.21 ± 0.32	4.85 ± 0.30	4.85 ± 0.24	5.21 ± 0.18
	28 days	5.65 ± 0.42	4.73 ± 0.34	4.21 ± 0.32	4.26 ± 0.26	4.81 ± 0.20
	42 days	3.48 ± 0.75	3.86 ± 0.81	5.21 ± 1.01	3.65 ± 0.44	3.92 ± 0.30
	56 days	4.78 ± 0.41	4.45 ± 0.28	3.79 ± 0.44	4.97 ± 0.46	5.02 ± 0.26
Creatinine (µmol/L) (R = 44.2–97.2)	Before	81.41 ± 10.00	45.98 ± 2.45	55.47 ± 8.97	81.87 ± 9.84	72.72 ± 9.22
	14 days	53.22 ± 2.15	51.74 ± 2.52	57.55 ± 3.26	55.01 ± 2.12	59.43 ± 2.21
	28 days	58.34 ± 2.04	55.88 ± 4.22	56.70 ± 3.71	58.91 ± 2.34	61.46 ± 1.22
	42 days	64.09 ± 2.37	59.95 ± 2.28	59.41 ± 4.11	59.56 ± 4.66	63.00 ± 1.83
	56 days	57.28 ± 4.05	51.19 ± 4.70	56.47 ± 5.65	48.64 ± 4.32	56.70 ± 4.03

TP=Total protein, ALB=Albumin, AST=Aspartate Aminotransferase

**Table 5:** Regression coefficients of blood biochemical parameters.

Biochemistry indicators	Time	Regression coefficient					Intercept	Time	Treatment × time

		A bolus	B bolus	Granules	N control	P control			
TP (g/L)	Before	0.30^NS^	−4.62^NS^	−4.49^NS^	Ref.	−2.22^NS^	55.95***	NS	NS
	14 days	−3.37^NS^	−4.18^NS^	−1.01^NS^	Ref.	0.20^NS^	61.35***		
	28 days	−1.51^NS^	−5.57^NS^	−5.95^NS^	Ref.	−1.08^NS^	60.98***		
	42 days	3.23^NS^	−2.19^NS^	−2.09^NS^	Ref.	2.41^NS^	56.22***		
	56 days	7.12^NS^	−6.25^NS^	1.61^NS^	Ref.	5.31^NS^	52.26***		
ALB (g/L)	Before	1.82^NS^	−6.76**	−6.94**	Ref.	−1.17^NS^	29.31***	NS	NS
	14 days	−0.35^NS^	−0.98^NS^	−0.40^NS^	Ref.	1.17^NS^	26.84***		
	28 days	0.05^NS^	−1.94^NS^	−2.98^NS^	Ref.	1.22^NS^	30.38***		
	42 days	1.74^NS^	−1.08^NS^	−0.96^NS^	Ref.	2.29^NS^	29.11***		
	56 days	3.07^NS^	−3.37^NS^	0.57^NS^	Ref.	4.15^NS^	27.76***		
AST (U/L)	Before	7.67^NS^	5.17^NS^	−3.50^NS^	Ref.	−3.33^NS^	85.33***	***	NS
	14 days	13.33*	11.83^NS^	0.67^NS^	Ref.	−0.17^NS^	83.67***		
	28 days	12.83^NS^	1.50^NS^	7.17^NS^	Ref.	−5.83^NS^	103.50***		
	42 days	29.83***	12.17^NS^	3.17^NS^	Ref.	9.83^NS^	58.67***		
	56 days	19.00^NS^	−8.17^NS^	3.83^NS^	Ref.	8.17^NS^	71.50***		
Urea (mmol/L)	Before	1.23**	−0.27^NS^	−1.00*	Ref.	−0.25^NS^	4.49***	NS	NS
	14 days	−0.90**	−0.64^NS^	−0.01^NS^	Ref.	0.35^NS^	4.85***		
	28 days	1.39***	0.47^NS^	−0.04^NS^	Ref.	0.55^NS^	4.26***		
	42 days	−0.17^NS^	0.22^NS^	1.57^NS^	Ref.	0.27^NS^	3.65***		
	56 days	−0.19^NS^	−0.52^NS^	−1.18**	Ref.	0.05^NS^	4.97***		
Creatinine (µmol/L)	Before	−0.46^NS^	−35.9***	−26.40**	Ref.	−9.14^NS^	81.86***	***	***
	14 days	−1.78^NS^	−3.27^NS^	2.54^NS^	Ref.	4.43^NS^	55.00***		
	28 days	−0.57^NS^	−3.03^NS^	−2.21^NS^	Ref.	2.55^NS^	58.91***		
	42 days	4.53^NS^	0.40^NS^	−0.15^NS^	Ref.	3.44^NS^	59.56***		
	56 days	8.64^NS^	2.55^NS^	7.83^NS^	Ref.	8.06^NS^	48.64***		

Ref.=Reference group, NS: Not significant,

* p < 0.1,

** p < 0.05,

*** p < 0.01. TP=Total protein, ALB=Albumin, AST=Aspartate aminotransferase

### FEC dynamics

**Figure 4:**
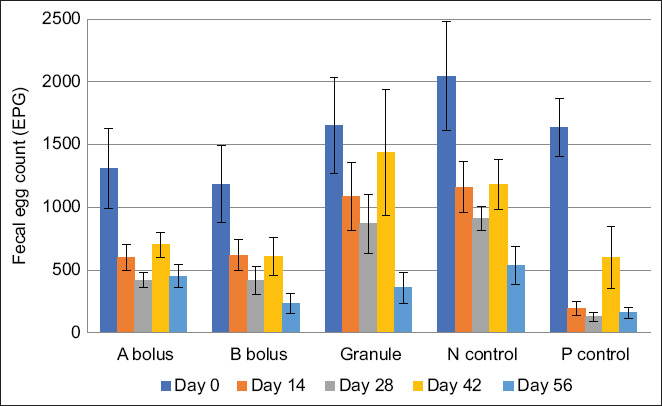
The mean number of strongylid eggs in fecal samples.

Initial FECs (day 0) confirmed high gastrointestinal strongylid burdens: Group A (1,366.7 EPG), B (1,125 EPG), granule (1,650 EPG), P control (1,633.3 EPG), and N control (2,046.7 EPG). By day 14, FECs had declined in all groups. P control maintained low levels thereafter. Groups A and B exhibited significant reductions to 625 and 591.7 EPG, respectively. The granule group reached 1,083.3 EPG, while the N control dropped to 1,158.3 EPG. By day 28, reductions continued: Group A (316.6 EPG), B (516.7 EPG), granules (866.7 EPG), and N control (908.3 EPG). An increase in FEC was noted in all groups on day 42. Final FECs on day 56 showed further decreases: Group A (358.3 EPG), B (325 EPG), granules (358.3 EPG), N control (533.3 EPG), and P control (158.3 EPG) (Figure 4).

## DISCUSSION

### Innovative delivery system for phytotherapeutics

This study evaluated the effects of freeze-dried *T. vulgare* leaf extract administered through granules and intraruminal boluses on gastrointestinal nematodes in sheep, assessing clinical health, weight gain, and hematological and biochemical parameters. A notable innovation was the use of HME to develop rumen-retentive boluses allowing sustained release in ruminants. Unlike conventional HME systems used in human pharmaceuticals, this study employed zone-specific low-temperature extrusion to preserve the bioactivity of plant compounds. The bolus design, incorporating a stainless-steel core and perforated cap, facilitated gradual disintegration over 1.5 months under simulated rumen conditions (pH 6.4–6.8, 38°C–41°C).

### Dose considerations and feed palatability

Phytotherapeutic agents hold promise in reducing gastrointestinal strongylid burdens in livestock. However, at elevated doses, these compounds may become toxic [19, 20]. Determining an optimal therapeutic window, balancing efficacy and safety, is essential. Tannins, commonly found in medicinal plants, promote productivity by limiting protein catabolism and enhancing immunity; however, they can negatively affect palatability and digestibility [21]. Literature suggests that tannin content above 50 g/kg dry matter can reduce intake [22] In this study, all groups gained weight, with the greatest gain in the control group. The granule group had the least gain, possibly due to the astringent taste of *T. vulgare*, which may have reduced feed intake despite the use of molasses.

### Hematological responses to treatment

High gastrointestinal strongylid loads impact erythrocytic indices such as RBC, HGB, and packed cell volume [23–26]. In this study, regression coefficients for RBC (p < 0.05) and HGB (p < 0.01) were elevated at baseline in Groups A and B. By day 28, intergroup hematological differences were no longer significant. WBC levels remained within normal ranges. Group B had significantly higher WBC regression coefficients than the N control on days 14 and 28 (p < 0.05), corroborating findings from Lima *et al*. [24]. On day 56, monocytosis was observed in three sheep in the granule group, potentially indicating intestinal inflammation or immunomodulatory responses triggered by *T. vulgare* [27].

### Biochemical changes and liver function

ALB and TP levels, often suppressed in severe nematode infections due to malabsorption and catabolism, were low at baseline across all groups [28, 29]. By day 28, values improved but remained lower in some groups. AST values were initially normal but dropped below physiological ranges by day 42 in the granule, negative control, and positive control groups. Group B maintained low AST levels by day 56. Similar AST reductions have been linked to liver regeneration, possibly due to the hepatoprotective effects of plant triterpenoids [30, 31].

### Mechanisms of anthelmintic action

Condensed tannins inhibit parasite development and feeding by disrupting their cuticle, while alkaloids may affect the parasite’s nervous system or gut motility [32–36]. Tannins also indirectly improve host immunity by protecting dietary proteins from ruminal degradation, increasing amino acid delivery to the duodenum. In this study, tannin-containing treatments improved weight gain and decreased FECs. Such effects have been observed by Besharati *et al*. [32], with reductions of 20%–50% in EPG in tannin-fed sheep.

### Parasitological outcomes and efficacy comparisons

All groups started with high strongylid infection levels (1,366–2,046 EPG) [36, 37]. By day 56, infection intensity decreased to moderate levels (325–533 EPG). Similar studies [12, 16, 19, 24, 30, 38, 39] using *Swertia chirata* or *A. absinthium* extracts showed EPG reductions from high to medium or low, depending on the plant, dose, and solvent used. In a study by Amirov *et al*. [12] using *T. vulgare*, the final EPG dropped to 67, despite the starting value already being low (185 EPG).

### Limited evidence for bolus-based herbal delivery

In our study, the extract was administered through bolus and granule forms. Most previous studies have used pure plant extracts or powders [40]. To date, no studies have evaluated intraruminal bolus delivery of phytotherapeutics in sheep, although similar systems exist for mineral supplementation [41, 42]. Our findings show the feasibility and safety of bolus use in sheep.

### Implications for future use and recommendations

The present study supports previous findings that plant-based agents exhibit anthelmintic activity and can be used preventively in ruminants [14, 42–45]. Our research confirms that intraruminal bolus administration is a practical, non-invasive method for veterinary use. While granules are more convenient for farmers, their palatability limits intake. Future research should investigate various formulations, dosages, and field trials under natural grazing conditions to validate and optimize the use of *T. vulgare* for sustainable parasite control.

## CONCLUSION

This study demonstrated that freeze-dried *T. vulgare* leaf extract, delivered through intraruminal boluses and granules, exerted measurable antiparasitic effects against gastrointestinal strongylids in sheep, while maintaining animal health and physiological stability. Notably, the bolus formulation, developed using a modified low-temperature HME process, proved effective in ensuring sustained release under rumen-simulated conditions and achieved greater reductions in FECs compared to granule administration. By day 56, FECs had declined significantly across all treatment groups, with the bolus groups (A and B) showing EPG reductions to 358.3 and 325, respectively, compared to 533.3 in the untreated control.

From a practical standpoint, the study confirms the feasibility of using herbal intraruminal boluses as a controlled-release platform for veterinary phytotherapy. The boluses were well tolerated and did not adversely affect hematological or biochemical parameters, indicating safety and potential for broader application. Moreover, this method offers a non-invasive, veterinarian-administered delivery route with minimized handling stress and improved compliance.

Strengths of the study include its novel approach to phytotherapeutic delivery, rigorous randomized double-blind design, and comprehensive monitoring of clinical, hematological, and parasitological indices. The use of zone-specific extrusion technology to preserve bioactivity and the simulation of rumen conditions for disintegration testing further enhances its translational relevance.

However, the study has limitations, including a relatively small sample size, a short observation period, and evaluation under controlled rather than natural grazing conditions. Additionally, variability in feed palatability may have influenced extract intake in the granule group, potentially affecting the efficacy outcomes.

Future research should focus on larger-scale field trials under varied ecological and management systems, longer follow-up periods to assess reinfection dynamics, and optimization of granule palatability. Comparative studies across other medicinal plants and combinations thereof could help identify synergistic effects and refine dosing strategies.

This study provides promising evidence supporting the development of *T. vulgare*-based intraruminal boluses as a sustainable alternative to synthetic anthelmintics in sheep. Such innovations align with One Health goals by reducing chemical use and promoting environmentally friendly parasite control strategies in ruminant production systems.

## DATA AVAILABILITY

All the generated data are included in the manuscript.

## AUTHORS’ CONTRIBUTIONS

LK, DK, DB, and IL: Conceptualization of the study, methodology, and drafted and revised the manuscript. DB and OR: Bolus design and 3D printing. DB, RT, AB, OB, KL, and OR: Technology and development of bolus composition and bolus dosage form. AV, AK, AM, and DK: Collected clinical data and performed parasitological analyses. IL: Statistical analysis. All authors have read and approved the final manuscript.
